# Myeloid Derived Suppressor Cells: Key Drivers of Immunosuppression in Ovarian Cancer

**DOI:** 10.3389/fimmu.2019.01273

**Published:** 2019-06-04

**Authors:** Thaïs Baert, Ann Vankerckhoven, Matteo Riva, Anaïs Van Hoylandt, Gitte Thirion, Gerhardt Holger, Thomas Mathivet, Ignace Vergote, An Coosemans

**Affiliations:** ^1^ImmunOvar Research Group, Laboratory of Tumor Immunology and Immunotherapy, Department of Oncology, KU Leuven, Leuven, Belgium; ^2^Department of Gynecology and Gynecologic Oncology, Kliniken Essen Mitte, Essen, Germany; ^3^Laboratory of Tumor Immunology and Immunotherapy, Department of Oncology, KU Leuven, Leuven, Belgium; ^4^Vascular Patterning Lab, Center for Cancer Biology, VIB, KU Leuven, Leuven, Belgium; ^5^PARCC, HEGP Institute (team 9), INSERM U970, Université Paris Descartes, Paris, France; ^6^Laboratory of Gynecologic Oncology, Department of Oncology, KU Leuven, Leuven, Belgium; ^7^Department of Gynaecology and Obstetrics, Leuven Cancer Institute, University Hospitals Leuven (UZ Leuven), Leuven, Belgium

**Keywords:** ovarian cancer, immunosuppression, myeloid derived suppressor cells, adaptive immune system, innate immune system

## Abstract

The presence of tumor infiltrating lymphocytes (TILs) is associated with a longer overall survival in advanced stage epithelial ovarian cancer. Despite the prognostic impact of TILs, response to checkpoint-inhibitors and antigen-specific active immunotherapy is limited in ovarian cancer. The goal of our study was to investigate the interaction between ovarian cancer and the innate and adaptive immune system in the ID8-fLuc syngeneic ovarian cancer mouse model. For the *in vivo* experiments C57BL/6, B6.129S7-Rag1^tm1Mom^/J, and B6.129P2(SJL)-Myd88^tm1.1Defr^/J mice were inoculated with ID8-fLuc. *In vivo* depletion experiments were performed using clodronate liposomes (CL), anti-CD8a, anti-GR1, anti-colony stimulating factor 1 (anti-CSF1), and TMβ1 (anti-CD122). Immune read out was performed by fluorescent activated cell sorting analysis for effector T cells, regulatory T cells, natural killer cells, B cells, macrophages, and myeloid derived suppressor cells (MDSC), immunohistochemistry for MDSC and tumor-associated macrophages (TAM) and immunofluorescence for M1 and M2 TAM in the vascular context. The effect of MDSC on T cell proliferation and phenotype were studied *in vitro*. We discovered that the absence of T and B cells did not influence tumor growth or survival of B6.129S7-Rag1^tm1Mom^/J mice compared to immunocompetent C57BL/6 mice. CL-induced macrophage depletion promoted tumor proliferation and shortened survival in C57BL/6 mice (*p* = 0.004) and in B6.129S7-Rag1^tm1Mom^/J mice (*p* = 0.0005). During CL treatment, we observed a clear increase of pro-inflammatory cytokines (*p* ≤ 0.02) and monocytic MDSC (*p* ≤ 0.01). Selective depletion of MDSC by anti-GR1 improved survival, certainly in comparison to mice treated with anti-CSF1 (*p* = 0.01—median survival 91 vs. 67.5 days). B6.129P2(SJL)-Myd88^tm1.1Defr^/J mice displayed to a longer median survival compared to C57BL/6 mice (90 vs. 76 days). MDSC activated by ID8-fLuc conditioned medium or ascites of tumor-bearing mice showed T cell suppressive functions *in vitro*. Based on these findings, we conclude that the adaptive immune system does not efficiently control tumor growth in the ID8-fLuc model. In addition, we discovered a prominent role for MDSC as the driver of immunosuppression in the ID8-fLuc ovarian cancer mouse model.

## Introduction

Ovarian cancer is the 5th leading cause of cancer death for women in developed countries (1). Standard treatment for advanced stage epithelial ovarian cancer is cytoreductive surgery in combination with platin-based chemotherapy (2). Despite radical surgery and excellent responses to first line chemotherapy, most patients diagnosed with advanced ovarian cancer do not survive beyond 5 years after diagnosis because of treatment-resistant recurrences (3). Ovarian cancer can be subdivided into four subtypes based on mRNA and miRNA expression, DNA copy number, DNA promotor methylation, and whole-exome DNA sequence analysis: immunoreactive, differentiated, proliferative, and mesenchymal (4). The immunoreactive subtype, which characterized by increased expression of CXCL11, CXCL10, and CXCR3, and displays the most favorable overall survival (OS) compared to the other subgroups (5). In line with this evidence, Zhang et al. demonstrated that the presence of tumor infiltrating lymphocytes (TILs) significantly correlates with improved survival in advanced epithelial ovarian cancer (6). In 2015, the Ovarian Cancer Action meeting suggested to study the interaction between ovarian cancer and the immune system, in order to develop strategies aimed at potentiating the anti-tumor immune response (7). Despite these efforts, only a limited number of ovarian cancer patients have responded to checkpoint-inhibitor therapy (8–10). In addition to this, no significant survival benefit was observed in ovarian cancer patients receiving antigen-specific active immunotherapy to date, most likely due to an overwhelming immunosuppression (11, 12).

Unlike the adaptive immune system, the innate immune system has not been extensively studied in the context of ovarian cancer, where it might be a key driver of immunosuppression. In previous studies, a high number of alternatively activated M2 tumor-associated macrophages (TAM) in ascites has been linked to poor clinical outcome. Furthermore, given the positive effects of anti- vascular endothelial growth factor (VEGF) treatment in ovarian cancer and the evidence that TAM are an important source of VEGF, targeting TAMs could also be interesting therapeutic option in this context (13–15). In addition, Cui et al. demonstrated that a high number of CD33^+^ cells in the tumor microenvironment was prognostic for shorter PFS (*p* = 0.006) and OS (*p* = 0.02) (16). The role of other innate immune cells, such as natural killer (NK) cells, dendritic cells, etc., remains unclear in ovarian cancer.

In this study, we discovered that depleting immune effector cells of the adaptive immune system (CD8^+^ T cells) does not increase tumor growth or influence survival in the ID8-fLuc model. We therefore explored the role of the innate immune system in the inhibition of the adaptive immune response. We observed a key role for (monocytic) myeloid derived-suppressor cells (mMDSC) in immune surveillance in the ID8-fLuc model.

## Materials and Methods

### Mice

Six- to eight-week-old mice were used. C57BL/6 and C57BL/6/BrDCHsd-Tyrc mice were obtained from Harlan/Envigo (Horst, Netherlands) or from an internal colony at KU Leuven. C57BL/6J-Tyrc-2J/J, B6.129S7-Rag1^tm1Mom^/J, and B6.129P2(SJL)-Myd88^tm1.1Defr^/J mice were obtained via Charles River from The Jackson Laboratory (Bar Harbor, ME, USA). For the *in vivo* experiment, only female mice were used. C57BL/6/BrDCHsd-Tyrc and C57BL/6J-Tyrc-2J/J are albino C57BL/6 mice, lacking all pigment from skin, hair and eyes.

B6.129S7-Rag1^tm1Mom^/J are immune deficient mice with a C57BL/6 background, lacking for mature T or B cells (17). B6.129P2(SJL)-Myd88^tm1.1Defr^/J are C57BL/6 mice that have a defect in the Myd88 cytosolic adapter, a protein which plays a central role in dendritic cells metabolism and in the immunosuppressive function of MDSC by activating NADPH oxidase and arginase-1 (18, 19).

Ovarian cancer was induced in the mice by intraperitoneal (IP) administration of 5 × 10^6^ ID8-fLuc cells dissolved in 100 μL cold Phosphate-Buffered Saline (PBS). The ID8-fLuc cell line was transducted by the Laboratory of Molecular Virology and Gene Therapy and Leuven Viral Vector Core in our institute. All *in vivo* experiments were performed with 5–6 mice per group and passages 2–4 of the ID8-fLuc cells. No systematic mycoplasma testing was performed. Severely ill animals were euthanized following humane endpoints as previously described by our group (20). All animals were housed and treated according to the Federation for Laboratory Animal Science Associations guidelines (21). Ethical approval was obtained from the local Ethical Committee (p075/2014 and p125/2017).

### Bioluminescence Imaging (BLI)

Non-invasive bioluminescence imaging (BLI) was used to evaluate tumor burden in albino C57BL/6/BrDCHsd-Tyrc and C57BL/6J-Tyrc-2J/J mice. As read-out, we used the maximum luminescence after administration of D-Luciferin (Promega, Madison, WI, USA) as a measure of viable tumor load. Image analysis was performed on the IVIS Spectrum Preclinical *in vivo* Imaging System (PerkinElmer, Waltham, MA, USA) at the Molecular Small Animal Imaging Centre (moSAIC) at the KU Leuven (22). The first scan was performed 1 week after tumor challenge in order to obtain a baseline of tumor engraftment. Subsequent measurements were performed once a week until 6 weeks after inoculation. In the CD8 T cell depletion experiment mice were scanned only scanned twice (week 1 and week 6 after tumor inoculation).

### *In vivo* Depletion Experiments

Clodronate Liposomes (CL) were purchased from Liposoma (Amsterdam, The Netherlands). We started treating the mice 1 week after tumor challenge with CL IP twice a week at a dosage of 0.05 mg/g bodyweight. As a control, PBS liposomes were used in preliminary experiments.

Depletion of CD8^+^ T cells was achieved using anti-CD8a (clone 53-6.72) purchased from BioXCell (West Lebanon, NH, USA). Three weeks after tumor inoculation, we administered a loading dose of 0.5 mg per mouse IP on 3 consecutive days after which we performed weekly maintenance IP injections of 1 mg in accordance to manufacturers' protocol.

For the depletion of NKp46^+^ NK cells we used TMβ1 (anti-CD122 monoclonal antibody), which was a kind gift of Ben Sprangers and Mark Waer (Lab of experimental transplantation, KU Leuven, Belgium). TMβ1 was produced in house by using the hybridoma technique. TMβ1 was administered IP at a dosage of 1 mg per mouse starting 1 day before tumor inoculation and continued at the same dosage twice a week.

Depletion of MDSC was achieved using anti-GR1 (Clone:RB6-8C5) purchased from BioXCell (West Lebanon, NH, USA). The monoclonal antibody was administered IP, at a dose of 10 mg/kg body weight, 3 times per week starting 1 week after inoculation.

A monoclonal antibody targeting colony stimulating factor 1 (CSF-1) (Clone:5A1) was used for the selective depletion of macrophages. Both the depleting antibody and the control antibody were bought from BioXCell (West Lebanon, NH, USA) and were administered IP. After a loading dose of 1 mg per mouse at day 21 after tumor challenge, a maintenance dose of 0.5 mg of anti-CSF1 or control antibody was administered once every 6 days IP.

### Immunohistochemistry (IHC)

Tumor tissue from metastatic disease was stained for the presence of Ly6C. In brief, paraffin-embedded tissue slices were deparaffinized and rehydrated using graded ethanol. Endogenous peroxidase activity was blocked by 0.5% H_2_O_2_ in methanol. After washing, heat-mediated antigen retrieval was carried out at 37°C in hydrogen chloride buffer containing pepsine 0.04% during 10 min. After cooling down and washing, non-specific binding was blocked and sections were incubated overnight at 4°C with rat anti-mouse Ly6C primary antibody (1:200 dilution; Thermo Fisher, Merelbeke, Belgium). After washing, sections were incubated during 30 min with goat anti-rat biotinylated secondary antibody (dilution 1:100; Abcam, Cambridge, UK), followed by another 30 min with streptavidin/peroxidase (dilution 1:1,000; DAKO/Agilent, Haasrode, Belgium). Staining was performed using 3,3′-diaminobenzidine (DAB) during 10 min. Sections were counterstained with Mayer's Hematoxylin solution, dehydrated with ethanol and mounted in DePex medium. Images were acquired on Zeiss Axio Scan.Z1 using a x20 objective and ZEN2 software (Zeiss). Four random fields at 20x magnification were chosen and used to manually count positive cells. The mean of the four values was used for downstream analyses. IHC was scored by AVK using Image J software [National Institutes of Health and the Laboratory for Optical and Computational Instrumentation (LOCI, University of Wisconsin)].

### Immunofluorescence Staining

Mice were sacrificed 33 days after tumor inoculation and peritoneal biopsies were taken. Tumor biopsies were prepared as 200 μm-thick vibratome sections, blocked and permeabilized in TNBT buffer [0.1 M Tris pH 7.4; NaCl 150 mM 0.5% blocking reagent from Perkin Elmer (Waltham, Massachusetts, USA), 0.5% Triton X-100] for 4 h at room temperature. Tissues were incubated overnight at 4°C with the following primary antibodies diluted in TNBT buffer: anti-glucose transporter-1 (Glut1) (Millipore, Burlington, Massachusetts, USA; 1:200 dilution), anti-Glut1 (Abcam, Cambridge, UK; 1:200 dilution), anti-major histocompatibility complex II (MHC-II) (Thermo Scientific, Waltham, Massachusetts, USA; 1:100 dilution) or anti-mannose receptor C type 1 (MRC1) (R&D Systems, Minneapolis, Minnesota, USA; 2 ug/ml). Next, slides were washed in TNBT buffer and incubated overnight at 4°C with the appropriate secondary antibody coupled with Alexa 488/555 (Life Technologies, Carlsbad, California, USA; 1:200 dilution) diluted in TNB Triton buffer. Tissues were washed and mounted on slides in fluorescent mounting medium (Dako/Agilent, Santa Clara, California, USA). Images were acquired using a Leica TCS SP8 confocal microscope. Semi-automated quantification analyses were performed using Fiji software (23).

### Immune Monitoring

The immune status of mice was evaluated at predefined time points, as described in the specific experimental set-ups. Mice were anesthetized with 80 μL ketamine [100 mg/mL; Nimatek (Eurovet, Bladel, Nederland)] and blood was collected from the retro-orbital plexus using glass capillaries. Blood was centrifuged at 8,000 rcf for 10 min. Serum was collected and stored at −80°C for further analysis. Next, the animals were euthanized by cervical dislocation. Peritoneal washing with 10 mL of PBS was performed to collect the circulating immune cells in ascites and from the peritoneal lining. Peritoneal washings were centrifuged for 5 min at 500 rcf and resuspended. Supernatant was collected and stored at −80°C for cytokine analysis. Using a Lymphoprep (Stemcell technologies, Vancouver, Canda) gradient, immune cells were isolated from the cell suspension and analyzed with flow cytometry (FACS).

Using flow cytometry, dead cells were excluded via eFluor780 fixable viability dye staining (Affymetrix Inc. San Diego, CA, USA). Immune cells were stained for myeloid cells, T cells and B cells using antibody panels, which are available as Supplementary Material (Supplementary Tables 1–3, respectively). For the myeloid panel, the cells were permeabilized using Leucoperm (Bio-Rad Laboratories Inc., Kidlington, UK) in accordance to manufacturers' protocol and stained for CD206. Permeabilization in the T cell panel was achieved using the eBioscience Foxp3/Transcription Factor Staining Buffer Set (ThermoFisher scientific, Waltham, Massachusetts, USA) and cells were then stained for FoxP3. Samples were acquired on the BD LSRFortessa (BD Biosciences, San Jose, CA, USA) and the analysis was performed using FlowJo Analysis software (Flow Jo, LLC, Ashland, Oregon, USA).

Cytokines in serum and ascites were determined using cytometric bead assay technique (BD Biosciences, San Jose, CA, USA). Both serum and peritoneal washings/ascites were used undiluted. The analysis was performed in accordance to the manufacturers' protocol using flex sets for IL-1 β, GM-CSF, IL-6, IL-10, MIP-1α, MIP-1β, TNFα, and IFNγ. Samples acquisition was performed on the BD LSRFortessa (BD Biosciences, San Jose, CA, USA) and the analysis was performed using FCAP Array Software v3.0 (BD Biosciences, San Jose, CA, USA).

### *In vitro* Experiments

MDSC were derived from bone marrow progenitor cells and splenocytes of C57BL/6 mice. Bone marrow progenitors, cells were isolated from bone marrow by flushing the long bones with PBS. For splenocytes, a single cell suspension was generated by passaging spleens through a 70 μm nylon strainer. From both splenocytes and bone marrow cells, dead cells were removed by the dead cell removal kit (130-090-101, Miltenyi Biotec, Bergisch Gladbach, Germany) in accordance to manufacturers' protocol. Next, MDSC were selected with the MDSC cell isolation kit (Miltenyi Biotec, Bergisch Gladbach, Germany), which provides two fractions based on relative GR1 expression: the MDSC-DIM corresponding to mMDSC and the MDSC-HIGH corresponding to gMDSC. For the T cell fraction, CD8^+^ T cells were selected from a single cell suspension of splenocytes using the CD8^+^ T cell isolation kit (Miltenyi Biotec, Bergisch Gladbach, Germany). T cells were activated by CD3/CD28 coated beads and cultured in medium supplemented with recombinant interleukin-2 (IL-2). Purity of all isolated cell types was verified by FACS.

In the first *in vitro* experiment naïve MDSC were exposed to ID8-fLuc conditioned medium. For this purpose, ID8-fLuc cells were grown in 96-well plates with trans well inserts (CoStar, Washington, D.C., USA), while MDSC were cultured in the inserts. Next the activated MDSC were co-cultured with CD8^+^ T cells. We evaluated the proliferation of T cells by quantification of the CFSE (Affymetrix Inc. San Diego, CA, USA) dilution.

In the second experiment MDSC were cultured in the presence of supernatant derived from ascites of tumor bearing mice to investigate the role of soluble factors in ascites. Subsequently, the stimulated MDSC were co-cultured with activated T cells and stained for FACS using the staining panel in Supplementary Table 4. Dead cells were excluded from the analysis by use of the eFluor780 fixable viability dye (Affymetrix Inc. San Diego, CA, USA). Cells were acquired on the BD Canto-II (BD Biosciences, San Jose, CA, USA). Analysis was performed using FlowJo Analysis software (Flow Jo, LLC, Ashland, Oregon, USA).

### Statistical Analysis

Prism 6 (GraphPad Software, Inc., San Diego, CA, USA) was used for statistical analysis and graphics. To evaluate statistical significance, α was set at 0.05. D'Agostino & Pearson omnibus normality test was used to evaluate normality. performed. For continuous variables, data are presented as mean ± SD or medians (interquartile ranges) as appropriate. Between-group comparisons used the Mann–Whitney *U*-test or *t*-test depending on the sample size for continuous variables. In cases more than two groups are compared one-way ANOVA test was performed, followed by Turkey's multiple comparison test if *p* < 0.05. Log-rank testing was performed to compare survival curves.

## Results

### Adaptive Immune Tolerance

**Figure 1:**
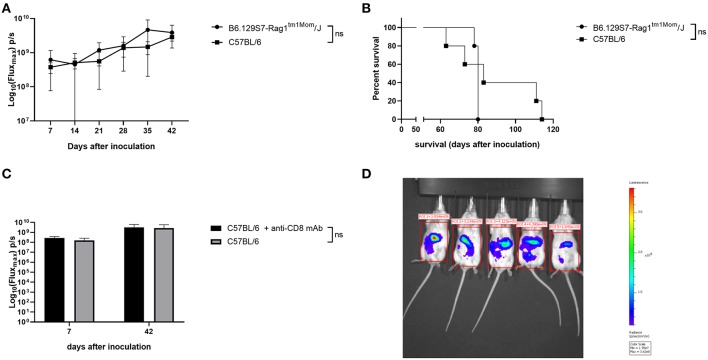
Lack of cancer immune surveillance by the adaptive immune system in the ID8-fLuc model. **(A)** Evaluation of tumor growth using BLI, Log_10_ transformation of maximal flux in photons per second (p/s) are shown as mean with standard deviation. We observed no significant (ns) difference in tumor growth in the mice lacking mature T cells and B cells (B6.129S7-Rag1^tm1Mom^/J) compared to the immunocompetent C57BL/6 mice. (*n* = 5 mice per group). **(B)** Kaplan-Meier curve showing the survival of B6.129S7-Rag1^tm1Mom^/J mice compared to immunocompetent C57BL/6 mice. Median survival is 80 days for B6.129S7-Rag1tm1Mom/J and 83 days for C57BL/6 (*n* = 5 mice per group). **(C)** Follow-up of tumor growth using BLI in wild type mice that received CD8 depletion (C57BL/6 + anti-CD8a mAb) compared to untreated mice (C57BL/6). Imaging was performed 1 and 6 weeks after tumor inoculation. No significant differences between the groups were observed. (*n* = 6 mice per group). **(D)** Representative picture of BLI imaging taken with the IVIS Spectrum Preclinical *in vivo* Imaging System.

We compared tumor growth of ID8-fLuc cells in B6.129S7-Rag1^tm1Mom^/J mice to tumor growth in C57BL/6 mice using BLI. As shown in Figure 1A, there was no significant difference in tumor burden between immunocompetent mice (C57BL/6) and mice lacking mature T and B cells (B6.129S7-Rag1^tm1Mom^/J). The B6.129S7-Rag1tm1Mom/J mice developed ascites at approximately the same moment as the C57BL/6 mice. There was no significant difference in survival between the two groups (Figure 1B). To investigate the specific role of CD8^+^ T cells in immune surveillance in the ID8-fLuc ovarian cancer model, we performed a depletion experiment by which we inoculated C57BL/6 mice with ID8-fLuc and started treating the mice with anti-CD8 20 days after tumor inoculation (onset of exponential tumor growth phase, as demonstrated earlier) (20) In this experiment, we did not observe a difference in tumor burden 6 weeks after inoculation between anti-CD8 treated and control mice, which corresponds to the results obtained with B6.129S7-Rag1^tm1Mom^/J mice (Figures 1C,D). Based on these findings, we can conclude that in the ID8-fLuc model the adaptive immune system has developed a tolerance against the tumor since knock-out or depletion of the adaptive immune system does not significantly influence tumor growth or survival. We therefore hypothesize that the innate immune system could play a role in rendering the effector cells of the adaptive immune system unfit for cancer immune surveillance in our model.

### Influence of Macrophages on Tumor Growth and Survival the ID8-fLuc Model

**Figure 2:**
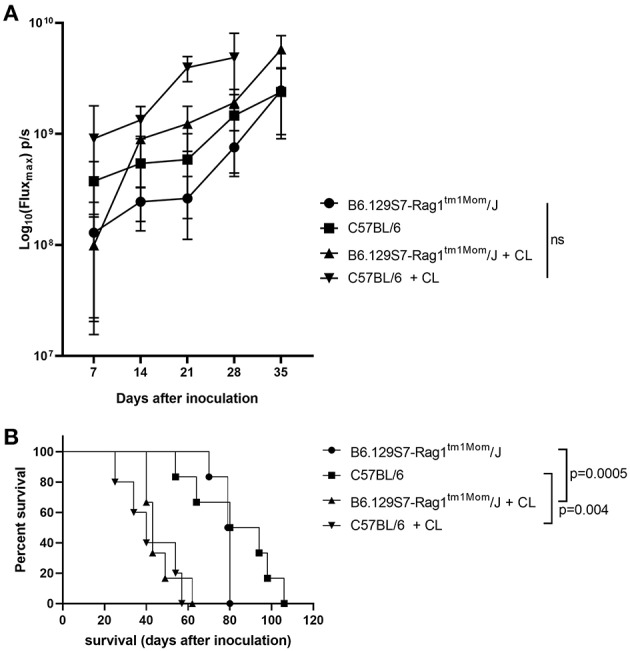
Influence of the innate immune system on tumor growth. **(A)** Evaluation of tumor growth using BLI, Log_10_ transformation of maximal flux in photons per second (p/s) are shown as mean with standard deviation. Tumor growth was increased in both in C57BL/6 mice and B6.129S7-Rag1^tm1Mom^/J mice when treated with clodronate liposomes (CL) (*n* = 6 mice per group). **(B)** Kaplan-Meier curve showing the survival of B6.129S7-Rag1^tm1Mom^/J mice and C57BL/6 mice treated with CL. In both groups treatment with CL led to a significant reduction in survival of the mice (*p* = 0.0005 and *p* = 0.004, respectively) (*n* = 6 mice per group).

In order to target innate immunosuppression, we treated the immunocompetent C57BL/6 model and in the B6.129S7-Rag1^tm1Mom^/J mice with CL (24). Compared to controls, the administration of CL led to a non-significant increase in tumor growth independent from the presence of T cells and B cells (Figure 2A), and to a significantly shorter survival of the mice (for C57BL/6 mice, *p* = 0.004; for B6.129S7-Rag1^tm1Mom^/J mice, *p* = 0.0005; Figure 2B). Administration of CL also reduced the incidence of ascites, both in B6.129S7-Rag1^tm1Mom^/J and in the C57BL/6 mice (16 and 33% of the B6.129S7-Rag1^tm1Mom^/J and C57BL/6 mice treated with CL developed ascites, respectively; in comparison to 90% of the untreated B6.129S7-Rag1^tm1Mom^/J and C57BL/6 mice) (20).

**Figure 3:**
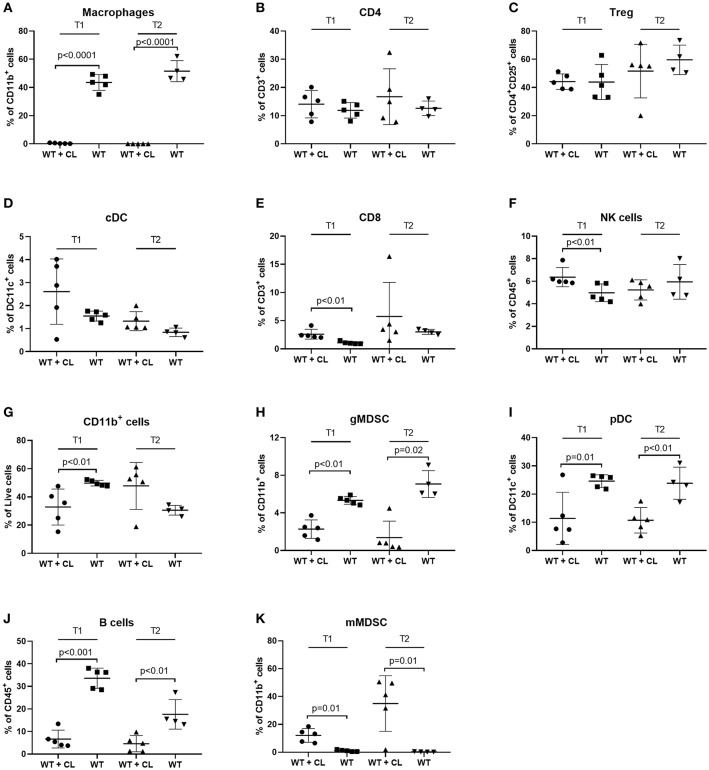
Effect of Clodronate liposomes (CL) on immune cells in the peritoneal cavity of tumor-bearing mice measured by FACS. Changes in immune cells in peritoneal washings during treatment with CL. Immunocompetent animals (C57BL/6) treated with CL (WT + CL) are compared to untreated wild type animals (WT) at two time points (T1 = 23 days after inoculation-T2 = 30 days after inoculation). (*n* = 5 mice per group). **(A)** Treatment with CL led to a relevant depletion of macrophages after treatment with CL to <1% of CD11b^+^ cells (*p* < 0.0001 for both time points). **(B–D)** No significant changes in CD4^+^ T cells, regulatory T cells (Treg) or conventional dendritic cells (cDC) were observed. **(E–G)** For CD8^+^ T cells, natural killer (NK) cells and CD11b^+^ myeloid cells significant differences [increase of CD8^+^ T cells and NK cells upon treatment with CL (*p* < 0.01 in both cases) and a reduction in CD11b^+^ cells in CL treated mice (*p* < 0.01)], were observed on the first time point only. **(H–J)** On both time points we observed a significant decrease in granulocytic myeloid-derived suppressor cells (gMDSC) (T1 *p* < 0.01-T2 *p* = 0.02), plasmacytoid dendritic cells (pDC) (T1 *p* = 0.01-T2 *p* < 0.01) and B cells (T1 *p* < 0.001-T2 *p* < 0.01). **(K)** Monocytic myeloid derived suppressor cells (mMDSC) increased after treatment with CL (*p* = 0.01 for both time points).

**Figure 4:**
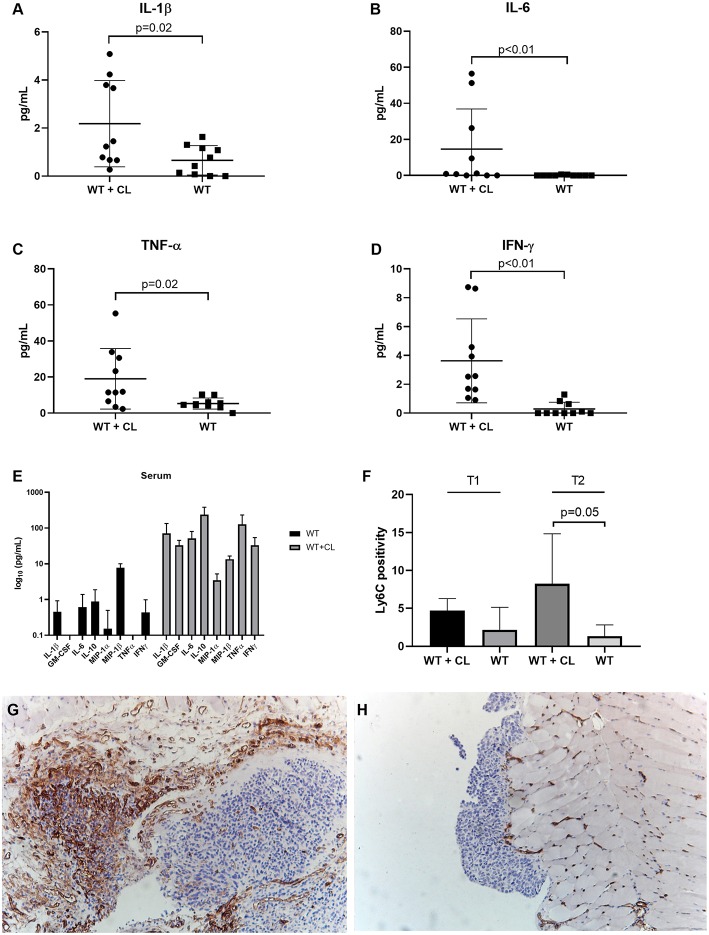
Effect of Clodronate liposomes (CL) on cytokines and tumor-infiltrating MDSC in tumor-bearing mice. Measurement of cytokines in peritoneal washings and serum of C57BL/6 mice treated with CL (WT + CL) are compared to untreated mice. **(A–D)** Changes in cytokines in peritoneal washings due to CL treatment: We observed a significant increase in pro-inflammatory cytokines such as IL-1β (Interleukin), IL-6, tumor necrosis factor-α (TNF-α), and interferon-γ (IFNγ). **(E)** Changes in cytokines in serum due to CL treatment at the second time point (day 30 after inoculation). We observed a significant increase in cytokines such as GM-CSF (*p* = 0.0004), IL-6 (*p* = 0.004), IL-10 (*p* = 0.006), Microphage inflammatory protein 1 (MIP1) α (*p* = 0.003), MIP1β (*p* = 0.009), and IFNγ (*p* = 0.008). **(F)** Using immunohistochemistry, we evaluated the percentage of Ly6C positivity during treatment with CL. Immunocompetent animals (C57BL/6) treated with CL (WT+CL) are compared to untreated wild type animals (WT). At the second time point, 30 days after inoculation we observed a significant higher number of Ly6C^+^ MDSC cells in the tumor upon treatment with clodronate liposomes (*p* = 0.05). (*n* = 5 mice per group). **(G,H)** Representative Ly6C staining of Immunocompetent animals (C57BL/6) treated with CL (WT+CL) **(G)** and untreated wild type animals (WT) **(H)** at the second time point. Magnification 10x.

Next, we studied the immunological changes during CL treatment to investigate the underlying mechanisms in detail. Using flow cytometry, we analyzed the immune cells present in peritoneal washings of C57BL/6 mice treated with CL and compared with PBS-treated controls at two predefined time points (T1 and T2, respectively, 23 and 30 days after tumor inoculation). Macrophages were reduced to < 1% of CD11b^+^ cells after the administration of CL, demonstrating they high efficacy of CL in depleting TAMs in the ID8-fLuc ovarian cancer model (Figure 3A). In accordance to literature, we observed no significant changes in CD4^+^ T cells, regulatory T cells (Treg) or conventional dendritic cells (cDC) following CL administration (Figures 3B–D) (25). At the first time point, we observed a higher amount of CD8^+^ T cells and NK cells in CL-treated mice; however, this effect was lost at the second time point (Figures 3E,F). The number of CD11b^+^ cells was significantly reduced upon treatment with CL at the first time point (Figure 3G). CL led not only to a significant decrease of TAM, but also a reduction in granulocytic MDSC (gMDSC), plasmacytoid DC (pDC), and B cells (Figures 3H–J). Monocytic MDSCs (mMDSC) were the only cell population, which were significantly increased at both time points upon CL treatment (Figure 3K). Additionally, we observed a clear significant increase in pro-inflammatory cytokines in ascites, such as interleukin-1β (IL-1β), IL-6 and tumor necrosis factor-α (TNFα), and interferon-γ (IFN- γ) (Figures 4A–D). This effect was not limited to ascites; we observed similar findings in serum of mice treated with CL (Figure 4E). As an additional readout, we performed IHC staining for Ly6C peritoneal biopsies of mice treated with CL or PBS. At the second time point, we observed an increase of Ly6C^+^ cells (*p* = 0.05), demonstrating the increased presence of intra-tumoral Ly6C^+^ MDSC upon CL treatment (Figures 4F–H).

### Selective Depletion of Innate Immune Cells Using Monoclonal Antibodies

**Figure 5:**
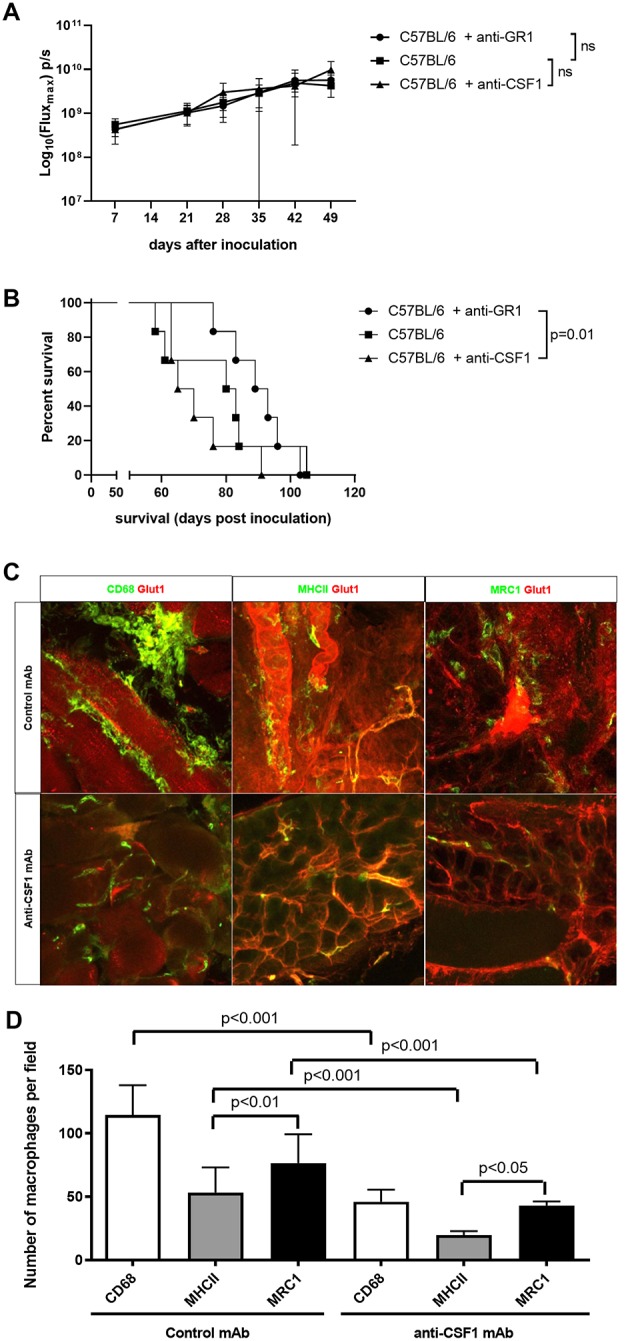
Selective depletion of innate immune cells using monoclonal antibodies (mAb). **(A)** Evaluation of tumor growth using BLI, Log_10_ transformation of maximal flux in photons per second (p/s) are shown as mean with standard deviation. We observed no significant differences in tumor load between the untreated immunocompetent mice (C57BL/6) and the MDSC-depleted mice (C57BL/6 + anti-GR1) or the macrophage depleted mice (C57BL/6 + anti-CSF1) (*n* = 6 mice per group). **(B)** Kaplan-Meier curve of untreated immunocompetent mice (C57BL/6) and the MDSC-depleted mice (C57BL/6 + anti-GR1) or the macrophage depleted mice (C57BL/6 + anti-CSF1). We observed a significantly improved survival in the mice treated with anti-GR1 compared to the mice treated with anti-CSF1 (*p* = 0.01). (*n* = 6 mice per group). **(C)** Immunofluorescent images of tumor biopsies of mice treated with anti-CSF1 or control mAb. In all panes blood vessels were stained for Glut1 in red. In the left pane, CD68 in green was used to stain total macrophages. In the middle pane, green MHC-II staining was used for M1 macrophages and on the right MRC1 staining in green was used for M2 macrophages. The images at the top represent the mice treated with the control antibody, while the images at the bottom represent the mice treated with anti-CSF-1 (scale bar: 50 μm) (*n* = 6 mice per group). **(D)** Quantitative evaluation of macrophages using immunofluorescent staining. Total macrophages were reduced to less than half due to anti-CSF1 (5A1). Both M1 and M2 macrophages were reduced in the same proportion following pan-macrophages mAb induced depletion (*n* = 6 mice per group).

Based on these findings, we performed a selective depletion of TAM, MDSC and NK cells using depleting monoclonal antibodies (mAb). In none of these experiments, we were able to detect significant differences in tumor growth using BLI (Figure 5A). Depletion of MDSC using anti-GR1 led to an increase in median survival from 81.5 to 91 days compared to untreated mice. The mice, which received anti-GR1, showed a significant survival advantage compared to the anti-CSF1 treated mice (*p* = 0.01; Figure 5B). Selective depletion of TAM using anti-CSF1 (5A1) led, similar to treatment with CL, to a non-significant reduction in median survival from 81.5 days (untreated mice) to 67.5 days (anti-CSF1 treated mice). Of note, treatment with anti-CSF1 depleted ~70% of TAM (Figures 5C,D), which was less profound (61.5% reduction of TAM after treatment with anti-CSF1 compared to the control antibody) compared to the depletion achieved by CL administration (near complete depletion of TAM). In addition, treatment with anti-CSF1 did not lead to a more favorable macrophage polarization (cytotoxic M1 vs. tumor supportive M2 ratio remained unchanged). Depletion of NK cells using anti-CD122 (TMβ1) did not influence tumor growth or survival of the mice.

### Ovarian Cancer (ID8-fLuc) Has a More Indolent Nature in B6.129P2(SJL)-Myd88tm1.1Defr/J Mice

**Figure 6:**
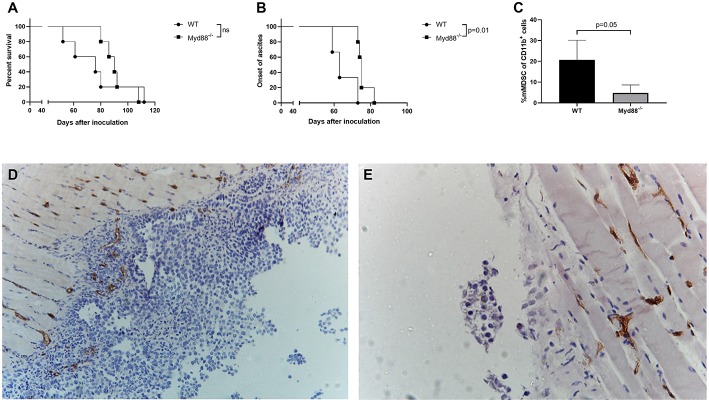
ID8-fLuc induced ovarian cancer has a more indolent nature in Myd88 knockout mice [B6.129P2(SJL)-Myd88^tm1.1Defr^/J] compared to wild type (C57BL/6). **(A)** The B6.129P2(SJL)-Myd88^tm1.1Defr^/J (Myd88^−/−^) mice had a median survival of 90 days compared to 76 days for C57BL/6 mice (WT). This difference in survival was not statistically significant. (*n* = 5 mice per group). **(B)** Onset of ascites was used here as a surrogate marker for onset of disease symptoms and here we observed a significant longer latency period. (*p* = 0.01) (*n* = 5 mice per group). **(C)** Using FACS we observed significantly less mMDSC in the B6.129P2(SJL)-Myd88^tm1.1Defr^/J mice compared to C57BL/6 mice. **(D,E)** Representative Ly6C staining of C57BL/6 **(D)** and B6.129P2(SJL)-Myd88^tm1.1Defr^/J **(E)** mice 8 weeks after inoculation. The C57BL/6 mice displayed macroscopically more peritoneal carcinosis compared to the B6.129P2(SJL)-Myd88^tm1.1Defr^/J mice. In addition, the peritoneal biopsies showed a higher Ly6C positivity in the tumor in the C57BL/6 mice compared to the B6.129P2(SJL)-Myd88^tm1.1Defr^/J mice.

In order to confirm our hypothesis that MDSC-mediated immunosuppression stimulates tumor growth and reduces survival in the ID8-fLuc model, we inoculated B6.129P2(SJL)-Myd88^tm1.1Defr^/J mice and C57BL/6 mice with ID8-fLuc cells. The goal of this experiment was to observe the *in vivo* effect of reduced MDSC-mediated immunosuppression. The Myd88 knock-out mice have a mutation in the Myd88 cytosolic adapter protein, which leads to an impaired immunosuppressive function of MDSC (18, 19). In these B6.129P2(SJL)-Myd88^tm1.1Defr^/J mice we observed a longer median survival after inoculation with ID8-fLuc compared to the wild type mice (C57BL/6) (90 days vs. 76 days, respectively) (Figure 6A). The B6.129P2(SJL)-Myd88^tm1.1Defr^/J mice also had a significant delay in the onset of ascites compared to C57BL/6 mice (75 days vs. 63 days, respectively,−*p* = 0.01) (Figure 6B). We also observed, in addition to the known reduced function of MDSC in Myd88^−/−^ mice, a significantly reduced presence of mMDSC in peritoneal lavage fluid of B6.129P2(SJL)-Myd88^tm1.1Defr^/J mice (Figure 6C). Using IHC we observed a larger tumor volume in wild type mice compared to the B6.129P2(SJL)-Myd88^tm1.1Defr^/J counterparts. In addition, we also found a reduced infiltration of Ly6C^+^ MDSC in the tumor of B6.129P2(SJL)-Myd88^tm1.1Defr^/J mice compared to C57BL/6 mice (Figures 6D,E). Based on these findings, we can conclude that MDSC support tumor growth and have a negative influence on survival of tumor-bearing mice.

Monocytic MDSC increase as tumor develops in ID8-fLuc model and suppress effector T cell functioning.

**Figure 7:**
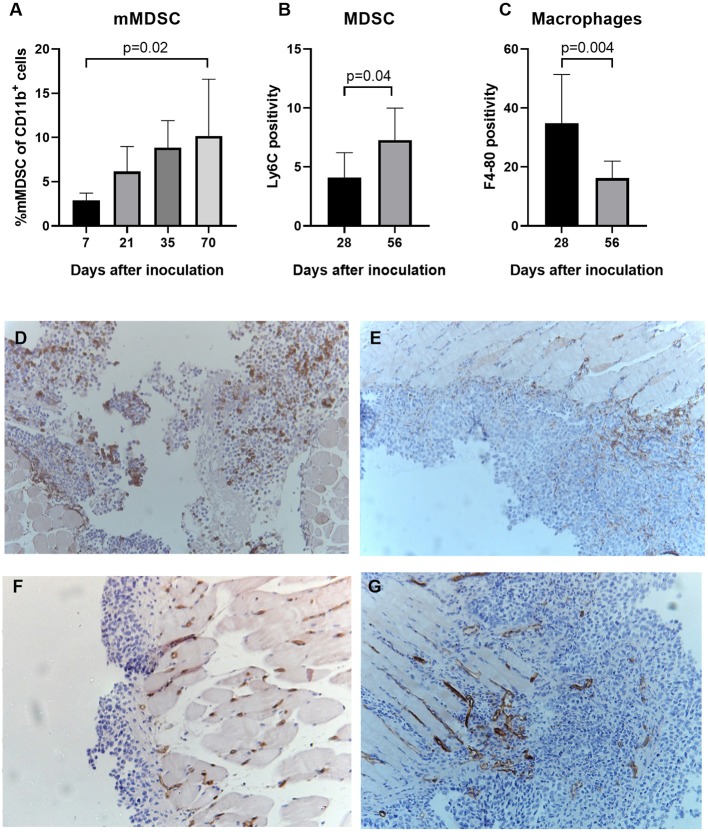
MDSC increase as tumor develops in ID8-fLuc model. **(A)** Using fluorescent activated cell sorting (FACS) we measured the relative number of mMDSC at different time points during tumor development. We observed a significant higher number of monocytic MDSC mice with end stage disease (10 weeks after tumor challenge) compared to mice with early stage disease (1 week after tumor challenge) (*p* = 0.02) (*n* = 3–6 mice per group). **(B,C)** Based on immunohistochemistry for F4-80 and Ly6C we evaluated the presence of, respectively, macrophages and MDSC in the tumor over time. **(B)** We observed a significant decrease in the number of total tumor-associated macrophages over time (*p* = 0.004). **(C)** Parallel to the results using FACS, we observed a significant higher number of Ly6C^+^MDSC 8 weeks after tumor challenge, compared to 4 weeks after tumor challenge (*p* = 0.04). **(D,E)** Representative images of the F4-80 staining 28 days **(D)** and 56 days **(E)** after inoculation. Magnification x10. **(F,G)** Representative images of the Ly6C staining 28 days **(F)** and 56 days **(G)** after inoculation. Magnification x10.

**Figure 8:**
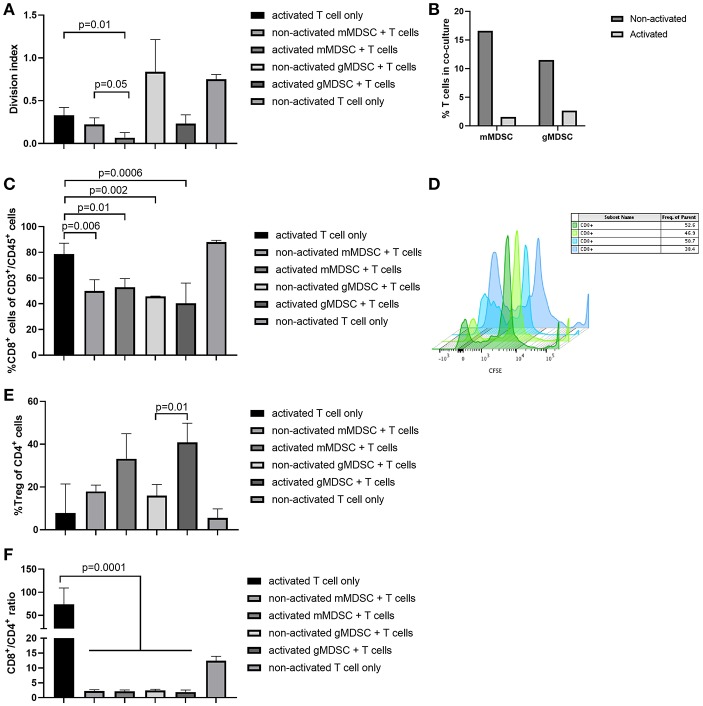
MDSC activated *in vitro* can suppress T cell proliferation and skew the T cell compartment toward a reduction in cytotoxic T cells. **(A)** mMDSC activated by conditioned medium of ID8-fLuc cell culture reduced T cell proliferation significantly (*p* = 0.05) as shown by carboxyfluoresceinsuccinimidyl ester (CFSE). **(B)** Acitvation of mMDSC and gMDSC by filtered ascites led to a strong reduction in the number of live T cells in co-culture. **(C)** FACS analysis of T cells co-cultured *in vitro* with MDSC. Co-culture of mMDSC and gMDSC led to a significant reduction of CD8 positivity. **(D)** MDSC activated by soluble factors in ascites induce a significant reduced T cell proliferation as shown by CSFE dilution. T cells in the presence of non-activated MDSC (blue) are capable of multiple divisions; in contrast T cells co-cultured with activated MDSC display a reduced proliferation (green). **(E)** Activation of gMDSC by filtered ascites led to an increase in regulatory T cells (Treg) (*p* = 0.01). **(F)** Co-culture of both mMDSC and gMDSC skewed the T cell phenotype toward CD4, which led to a reduction in the CD8^+^/CD4^+^ T cells ratio even before activation of MDSC (*p* = 0.0001).

Next, we studied the natural evolution of MDSC in the ID8-fLuc ovarian cancer model by assessing the relative numbers of MDSC in ascites over time in tumor-bearing mice and healthy controls. As anticipated from literature, we observed higher numbers of mMDSC in tumor-bearing mice compared to naive mice (26). Additionally, we observed significantly more mMDSC in ascites of mice with end stage disease compared to early stage disease (*p* = 0.02) (Figure 7A). Using immunohistochemistry, we observed an absolute reduction in TAM (*p* = 0.004) and an increase in absolute number of Ly6C^+^ MDSC (*p* = 0.04) in the tumor over time (Figures 7B–G). To study the immunological role of mMDSC in ovarian cancer further, we performed *in vitro* experiments. In these experiments, we evaluated the T cell suppressive capacities of MDSC after stimulation by soluble factors derived from ID8-fLuc cell culture or ascites. Activation of mMDSC by conditioned medium of ID8-fLuc cell culture reduced T cell proliferation (*p* = 0.05), as measured by CFSE (Figure 8A). Both mMDSC and gMDSC reduced the number of T cells in co-culture when activated by filtered ascites of tumor bearing mice (Figure 8B). Next, we explored the suppressive effect of MDSC on the different T cell subsets using FACS. Co-culture of T cells with mMDSC and gMDSC, led to a reduction in the percentage of CD8^+^ T cells in the T cell population, even without activation of the MDSC (Figures 8C,D). In addition, the number of regulatory T cells (Treg) increased during co-culture with activated gMDSC (Figure 8E). Co-culture of T cells with MDSC also led to a strong reduction in de CD8^+^/CD4^+^ T cell ratio, irrespective of the activation status of the MDSC (Figure 8F). Based on these *in vitro* experiments we can conclude that MDSCs activated by soluble factors present in ascites of ID8-fluc tumor bearing mice induced an unfavorable immune profile with increased regulatory T cells and decreased effector T cells.

## Discussion

In this paper we studied the interaction between ovarian cancer and the immune system in the ID8-fLuc ovarian cancer mouse model. In short, we demonstrated that tumor growth and survival of tumor bearing mice is not controlled by the adaptive immune system in the ID8-fLuc model. Tumor growth in B6.129S7-Rag1^tm1Mom^/J mice, which lack T cells and B cells, was similar to tumor growth in immunocompetent C57BL/6 mice. Survival did not significantly differ between both mice strains. Additionally, depletion of CD8^+^ T cells did not significantly influence tumor growth in the ID8-fLuc model. There are two main possible explanations for these findings. The first being lack of immunogenicity of the model itself. This is unlikely as multiple studies have shown the antigenicity and immunogenicity of the ID8 model (27–29). Therefore, we hypothesized that the adaptive immune system in the ID8-fLuc model could be rendered anergic. As the behavior of the tumor was very similar in both the specific CD8+ T cell depletion and the B6.129S7-Rag1^tm1Mom^/J mice, we hypothesized that the innate immune system might play a role in the immunosuppression exerted on the adaptive immune system.

In order to study the role of the innate immune system, macrophages more specifically, we explored the effect of CL in the ID8-fLuc model. Treatment with CL led to a shorter survival both in C57BL/6 mice as in B6.129S7-Rag1^tm1Mom^/J mice. As CL is considered a dirty drug, which effects are not limited to macrophages only, we investigated the effect of CL treatment on the immune system in the ID8-fLuc model. CL effectively depleted macrophages in the peritoneal cavity of tumor bearing mice. In addition to his, we observed a significant increase in mMDSC and proinflammatory cytokines, which might explain the poor survival of mice treated with CL. We hypothesize that the strong reduction in TAM (to <1% of CD11b^+^ cells) disrupts the homeostasis of the tumor microenvironment in the ID8-fLuc model. The observed cytokine reaction could explain the increase in mMDSC, since IL-6 is a known inducer of mMDSC expansion in humans and IL-1b correlates with mMDSC in blood of ovarian cancer patients (30, 31). We also observed a significant increase in Microphage inflammatory protein 1 (MIP1) α and MIP1β, which might have contributed to the recruitment of highly immunosuppressive CCR5^+^mMDSC (32). The activation of MDSC can lead to an increase in IL-6, IL-10, IL-1β, and IFNγ, creating a feedback loop (32). Based on the assumption that the rise in MDSC caused by the depletion of macrophages by CL was responsible for the detrimental effect on survival of the mice, we performed a more selective depletion experiment. We compared survival and tumor growth of mice treated with anti-CSF1 (selectively TAM depletion), anti-GR-1 (depletion of MDSC), and untreated tumor bearing mice. Selective reduction of GR-1^+^ MDSCs led to a small survival benefit, as was demonstrated previously by others (33). Survival of mice treated with anti-GR-1 was significantly longer than survival of anti-CSF1 treated mice. Depletion of macrophages by anti-CSF1 was less efficient compared to depletion achieved by CL, which might explain why the effect of anti-CSF1 on survival and tumor growth is less pronounced compared to the CL. To support our hypothesis that MDSC have a negative impact of survival on tumor bearing mice in the ID8-fLuc model, we induced ovarian cancer in B6.129P2(SJL)-Myd88^tm1.1Defr^/J mice. These mice carry a deletion of exon 3 of the myeloid differentiation primary response gene 88 locus, which leads to a reduced (immunosuppressive) function of MDSC. Median survival was longer in the B6.129P2(SJL)-Myd88^tm1.1Defr^/J mice compared to wild type C57BL/6 mice. In addition, onset of disease symptoms (ascites) was significantly delayed in the B6.129P2(SJL)-Myd88^tm1.1Defr^/J mice, supporting our hypothesis.

Next, we investigated the immunosuppressive effects of MDSC on T cells in the context of the ID8-fLuc model *in vitro*. In these experiments, we demonstrated that mMDSC and gMDSC activated by conditioned medium of ID8-fLuc cell culture or filtered ascites, led to a reduction in T cell proliferation and reduced the relative number of effector T cells in co-culture. These findings are supported by Horikawa et al. who demonstrated that MDSC suppress the CD8 T cells in the tumor microenvironment (34), suggesting that MDSC-induced immunosuppression might be one of the drivers of adaptive immunetolerance in ovarian cancer.

It should be noted, however that in an attempt to study the interaction between ovarian cancer and the immune system in a comprehensive way, we decided to use relatively nonspecific tools such as clodronate liposomes and B6.129S7-Rag1^tm1Mom^/J mice. As immunocompetent models for ovarian cancer are scarce, we limited ourselves to the ID8-fLuc model, this is of course also a possible bias. However, we used different methods, which all pointed toward an important role for (monocytic) MDSC in tumor-associated immunosuppression. In addition, the importance of the innate immune system, MDSC in particular, as a source of immunosuppression is being increasingly recognized in ovarian cancer. Cui et al. were the first to demonstrate a prognostic role for intra-tumoral MDSC in ovarian cancer (16, 34).

In addition, our study underscores the plasticity of the innate immune system and the balanced relationship between the different innate immune cells. A large part of the tumor stroma consists of TAM; therefore, it is not surprising that rash depletion of TAM, leads to a cytokine reaction, which attracts other innate cells to fill this niche. It is also important to note that macrophages and mMDSC originate from the same immature myeloid cells in bone marrow and that mMDSC can differentiate into macrophages (35). Therefore, it is not surprising that such interaction between the innate immune cells exist. Upon treatment with CL mMDSC were attracted to the tumor microenvironment, which led to worse survival of the mice, probably due to a detrimental effect on tumor immune control.

Until recently, tumor immunology research in ovarian cancer has focused mainly on the influence of the adaptive immune system on antitumor immunity (36). Only a limited number of studies have investigated the role of the innate immune system in ovarian cancer. We are the first to demonstrate that the presence of T cells was irrelevant for tumor growth and survival in the ID8-fLuc model. These results suggest that immunosuppression dominates the adaptive immune response in the ID8-fLuc model. In addition, we showed that MDSC are an important source of immunosuppression in ovarian cancer. This is an important finding as clinical immune oncology trials in ovarian cancer are currently focusing on the adaptive immune system. In ovarian cancer, the success has currently been limited to a small number of patients. Targeting MDSC might be a possible strategy to increase the number of patients who respond to immunotherapy. Preclinical studies have detected several possible strategies to deplete or inhibit MDSC, e.g., gemcitabine, 5-FU, ATRA, sunitinib, aspirin etc. (37). However, we believe that further preclinical and translational research is needed to design rational immunotherapeutic approaches in ovarian cancer.

## Contribution to the Field Statement

Ovarian cancer is the second most lethal type of gynecological cancer in women with an incidence rate of 12.5 per 100,000 women. Standard therapy consists of extensive surgery in combination with chemotherapy. As tumor-infiltrating lymphocytes have a positive prognostic impact in ovarian cancer, immune checkpoint inhibitors have been put forward as a new treatment modality. However, response was only 10% in monotherapy. According to our findings, this might be explained by underlying immune biology in ovarian cancer. In this paper, we demonstrate that the adaptive immune system is unable to control tumor growth in an ovarian cancer mouse model. We hypothesized that the innate immune system suppresses the adaptive immune response. We show that myeloid-derived suppressor cells (MDSC), increase during the disease course and that MDSC are able to suppress the T cells of the adaptive immune system. In addition, we show that suppression of MDSC function positively influences survival of mice with ovarian cancer. Therefore, we argue that MDSC play an important immunosuppressive role in ovarian cancer and that future studies on immunotherapy should consider combining agents that optimize the T cells response to strategies targeting innate immunosuppression.

## Data Availability

All datasets generated for this study are included in the manuscript and/or the Supplementary Files.

## Ethics Statement

All animals were housed and treated according to the Federation for Laboratory Animal Science Associations guidelines (21). Ethical approval was obtained from the local Ethical Committee at KU Leuven (p075/2014 and p125/2017).

## Author Contributions

All authors helped write and approved the submitted version of the manuscript. TB designed the study, wrote the manuscript, set-up and performed the *in vivo* experiments, analyzed the data, and performed the statistical analyses. AV helped with the *in vivo* experiments, performed *in vitro* experiments, and scored the IHC. MR provided technical assistance. GT and AVH performed FACS staining and acquisition. TM performed immunofluorescent staining. IV added to the concept of the study. AC supervised the experiments, helped write the manuscript, and conceived the study.

### Conflict of Interest Statement

The authors declare that the research was conducted in the absence of any commercial or financial relationships that could be construed as a potential conflict of interest.
