# Supplementary material for: Myeloid Derived Suppressor Cells: Key Drivers of Immunosuppression in Ovarian Cancer
Source: Front Immunol. 2019 Jun 4;10:1273. doi: 10.3389/fimmu.2019.01273 (PMC6558014; doi:10.3389/fimmu.2019.01273)
Supplement: Supplementary file 1 [file Table_1.DOCX]

### Supplementary tables

Supplementary table 1. *Myeloid panel*

| **Monoclonal Ab** | **Fluorophore** | **Supplier** |
| --- | --- | --- |
| CD11b | PerCPCy 5.5 | BD Biosciences (San Jose, CA, USA) |
| CD11c | AF700 | BD Biosciences (San Jose, CA, USA) |
| B220 | V500 | BD Biosciences (San Jose, CA, USA) |
| CD8a | PE CF595 | BD Biosciences (San Jose, CA, USA) |
| Ly6C | AF647 | BioLegend (San Diego, CA, USA) |
| Ly6G | FITC | BD Biosciences (San Jose, CA, USA) |
| CD193/CCR3 | PE | eBioscience (San Diego, CA, USA) |
| F4/80 | BV421 | BD Biosciences (San Jose, CA, USA) |
| I-A/I-E | BV650 | BD Biosciences (San Jose, CA, USA) |
| CD206/MMR | PE-Cy7 | eBioscience (San Diego, CA, USA) |

Supplementary table 2. *T cell panel*

| **Monoclonal Ab** | **Fluorophore** | **Supplier** |
| --- | --- | --- |
| CD45 | AF700 | eBioscience (San Diego, CA, USA) |
| CD3 | BV510 | BD Biosciences (San Jose, CA, USA) |
| CD4 | PerCP Cy5.5 | eBioscience (San Diego, CA, USA) |
| CD8 | BV421 | BD Biosciences (San Jose, CA, USA) |
| CD25 | PE | BD Biosciences (San Jose, CA, USA) |
| FoxP3 | AF488 | BD Biosciences (San Jose, CA, USA) |

Supplementary table 3. *B cell panel*

| **Monoclonal Ab** | **Fluorophore** | **Supplier** |
| --- | --- | --- |
| CD45 | AF700 | eBioscience (San Diego, CA, USA) |
| CD3 | BV510 | BD Biosciences (San Jose, CA, USA) |
| CD19 | AF647 | BD Biosciences (San Jose, CA, USA) |
| CD20 | PE | eBioscience (San Diego, CA, USA) |
| NK1.1 | BV421 | BD Biosciences (San Jose, CA, USA) |
| NKp46 | PerCP Cy5.5 | BioLegend (San Diego, CA, USA) |

Supplementary table 4.*T cell panel in vitro experiments*

| **Monoclonal Ab** | **Fluorophore** | **Supplier** |
| --- | --- | --- |
| CD45 | APC | eBioscience (San Diego, CA, USA) |
| CD3 | APC eFluor780 | eBioscience (San Diego, CA, USA) |
| CD4 | PerCP Cy5.5 | eBioscience (San Diego, CA, USA) |
| CD8 | BV421 | BD Biosciences (San Jose, CA, USA) |
